# Intranasal inoculation of sows with highly pathogenic porcine reproductive and respiratory syndrome virus at mid-gestation causes transplacental infection of fetuses

**DOI:** 10.1186/s13567-015-0283-z

**Published:** 2015-12-29

**Authors:** Tongtong Wang, Xiaofei Wang, Xin-an Li, Li Nie, Minxia Zhang, Sidang Liu, Xiaomin Zhao, Yingli Shang, En-min Zhou, Julian A. Hiscox, Yihong Xiao

**Affiliations:** Department of Basic Veterinary Medicine, Shandong Provincial Key Laboratory of Animal Biotechnology and Disease Control and Prevention, College of Veterinary Medicine, Shandong Agricultural University, Taian City, Shandong China; College of Veterinary Medicine, Northwest A&F University, Yangling, Shaanxi China; Institute for Immunology and School of Medicine, Tsinghua University, Beijing, China; Department of Infection Biology, Institute of Infection and Global Health, University of Liverpool, Liverpool, UK

## Abstract

Transplacental infection plays a critical role in the reproductive failure induced by porcine reproductive and respiratory syndrome virus (PRRSV), yet exposure of sows and gilts to classical PRRSV generally leads to reproductive failure after 85 days of gestation. We report, for the first time, that the susceptibility of fetuses to highly pathogenic PRRSV (HP-PRRSV) is similar at 60 days and 90 days of gestation. This difference from classical PRRSV may contribute to its high pathogenicity. A field study of the HP-PRRSV vaccine in pregnant sows at mid-gestation should be considered.

## Introduction, methods and results

Porcine reproductive and respiratory syndrome (PRRS), caused by porcine reproductive and respiratory syndrome virus (PRRSV), is one of the most severe swine diseases leading to great economic losses worldwide. PRRSV is an enveloped, single-stranded positive-sense RNA virus, belonging to the family *Arteriviridae* in the order *Nidovirales*. The genome of PRRSV is approximately 15 kb in length and contains at least 10 open reading frames encoding 8 structural proteins and 13–16 nonstructural proteins (nsps) [1, 2]. The *nsp2* gene is the most highly variable region of the genome and contributes to the virulence and genotype of PRRSV [3, 4]. In 2006, a highly pathogenic PRRSV (HP-PRRSV) was identified in China, causing great concern in the global swine industry [5]. HP-PRRSV is genetically characterized by a unique discontinuous 30-amino-acid deletion in *nsp2* gene [6, 7]. HP-PRRS caused by HP-PRRSV is characterized by high fever (41–42 °C), high illness rates (50–100%), high death rates (20–100%), and high abortion rates (40%), resulting in devastating economic losses [5–7]. To date, HP-PRRSV has been found in North, East, and South Asia [8–10].

Reproductive failure is one of the prominent clinical manifestations of PRRS, and pregnant sows exposed to PRRSV give birth to dead, stillborn, or mummified fetuses. Persistent uterine infection contributes to reproductive failure in sows. The ability of the virus to cross the placental barrier is critical for fetal infection because pig embryos are not susceptible to PRRSV prior to implantation [11, 12]. The ability to cross the placenta is strain-dependent; for example, the Spanish PRRSV strain 5710 has been shown to infect 20-day-old embryos when gilts were exposed at the onset of gestation [13]. In contrast, sows infected with a Danish isolate of PRRSV had the highest transplacental transmission rate at day 85 of gestation, with less transmission at day 72 of gestation, and none on day 45 of gestation [14]. Furthermore, it has been reported that a Danish vaccine strain (19407B) of PRRSV caused congenital infection, fetal death, and pre-weaning piglet mortality after it was administered intranasally to sows [15]. Another two European-type modified-live-virus vaccine strains, VP-046 Bis and All-183, can also cross the placenta and result in congenital infection of piglets at birth [16]. The typical type II isolate ATCC VR-2332 causes reproductive failure in 93-day-pregnant sows but does not induce reproductive failure when administered at mid-term gestation [17, 18]. Although the capacity for transplacental infection varies, classical PRRSV cause transplacental infection with consequent abortion predominantly when the sows are exposed during late gestation. However, it is unclear whether the newly emerged HP-PRRSV has an enhanced ability to cross the placenta.

To investigate the ability of HP-PRRSV to cross the placenta, 30 cross-bred Landrace × Large White gilts were obtained from a PRRSV-free herd and tested using both RT-PCR and enzyme-linked immunosorbent assay to confirm that they were PRRSV-negative. The gilts were artificially inseminated and randomly allocated to one of two control groups (*n* = 6 sows/group) or one of two treatment groups (*n* = 9 sows/group). Inoculums from uninfected cell cultures were used to sham-infect the two control groups at 60 and 90 days of gestation, respectively. The two treatment groups were inoculated intranasally at day 60 and 90 of gestation, respectively, with 10^6^ median tissue culture infective doses (TCID_50_) of HP-PRRSV strain TA-12 collected from HP-PRRSV infected Marc-145 cells (GenBank HQ417620). Clinical signs of PRRSV infection, including reduced appetite, low energy, and high fever, were observed from 3 days post infection (dpi). Two sows in the day 90 infected group aborted at 13 dpi and 17 dpi, corresponding to gestation days 103 and 107, respectively. All the sows in the sham-inoculation control groups remained clinically normal.

**Figure 1:**
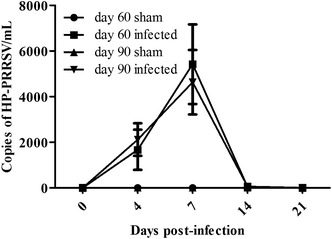
**Analysis of viremia in serum samples.** Viral load, measured as the copy number of highly pathogenic porcine reproductive and respiratory syndrome virus (HP-PRRSV), in serum samples from the day 60 and 90 infected groups at 0, 4, 7, 14, and 21 days post-infection (dpi). All samples were analyzed in triplicate.

A quantitative RT-PCR (qRT-PCR) targeting the HP-PRRSV conserved nucleocapsid (N) protein gene was developed to detect HP-PRRSV using specific primers (forward primer 5′-AGATCATCGCCCAACAAAAC-3′; reverse primer 5′-GACACAATTGCCGCTCACTA-3′). HP-PRRSV was detected in the sera from 4 to 14 dpi with qRT-PCR, with the peak titer at 7 dpi. No PRRSV mRNA were detected in the sera of the sham-infected sows throughout the experiment (Figure 1).

**Figure 2:**
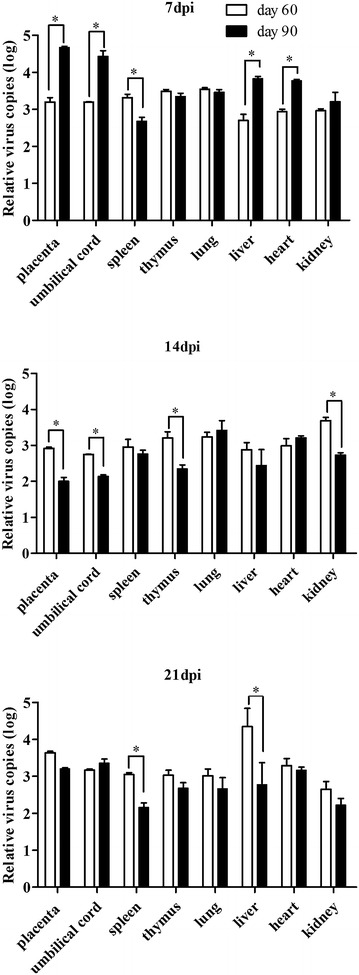
**HP-PRRSV loads in the organs at 7, 14, and 21 dpi.** All values are expressed as mean ± standard deviation of the mean (SD). Statistical analyses were performed using GraphPad Prism 5. Differences were considered significant at *P* < 0.05. * *P* < 0.05.

At 7, 14, and 21 dpi, three sows in the HP-PRRSV-infected groups and two sows in the sham-infected groups were sacrificed by barbiturate overdose, which is a euthanasia method approved by the Animal Care and Use Committee of Shandong Agricultural University. Tissue and blood samples were collected from the sows and fetuses. At necropsy, similar gross lesions were found in the day 60 and 90 infected sows, including interstitial pneumonia and lymph node edema. Sporadic hemorrhagic spots were also found in the fetal organs, including the liver, lymph nodes, and hearts of fetuses in both day 60 and 90 infected groups. In contrast, no gross lesions were found in the sham-infected sows or their fetuses. HP-PRRSV RNA was detected in the amnion and in the umbilical cords of infected sows. At 7 dpi, significantly higher levels of virus were detected in the amnion in sows infected at day 90 of gestation than in sows infected at day 60 of gestation (Figure 2). However, more copies of HP-PRRSV were detected in both the amnion and umbilical cords in day 60 infected sows than in day 90 infected sows at 14 dpi. These results indicate that the intranasal inoculation with HP-PRRSV led to successful infection of the sows.

**Figure 3:**
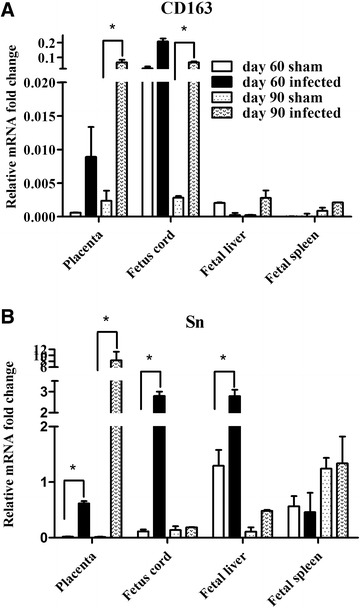
**Relative placental CD163 and Sn mRNA expression levels.** CD163 (**A**) and Sn (**B**) mRNA expression levels in day 60 and 90 infected and sham-infected sows at 7 dpi. All values are expressed as mean ± standard deviation of the mean (SD). Statistical analyses were performed using GraphPad Prism 5. Differences were considered significant at *P* < 0.05. * *P* < 0.05.

Sialoadhesin (Sn) and CD163 are PRRSV cellular receptors [19]. PRRSV has a restricted tropism for Sn^+^CD163^+^ macrophages [20, 21] and the presence of Sn^+^CD163^+^ macrophages in the placenta is essential for the transmission of the virus from mother to fetus. Thus, we evaluated placental relative mRNA expression levels of Sn and CD163 (compared with mRNA expression levels of beta actin) at 7 dpi (Figure 3). Two-way analysis of variance (ANOVA) performed using GraphPad Prism 5 revealed significant increases in the placental expression of Sn mRNA after HP-PRRSV infection at both day 60 and 90 of gestation. Placental expression of CD163 mRNA increased only in sows infected at day 90 of gestation.

To exclude the possibility that fetal infection was influenced by differences in sow susceptibility to the virus, the viral loads in the spleens, brains, lungs, and uteri of infected and sham-infected sows at 7 dpi were determined using qRT-PCR. There were no differences in the viral loads among any of the organs examined (data not shown), which suggests that we successfully developed an animal model suitable for the study of transplacental infection by HP-PRRSV.

Conventional RT-PCR targeting the *nsp2* gene was employed to assess infection status. Detection of the 680-bp *nsp2* gene is more specific than detection of the *N* gene because it is less abundantly expressed. The fetal infection status was evaluated by detecting the *nsp2* gene in fetal spleens. Total splenic RNA were extracted with TRIzol Reagent (Life Technologies, Gaithersburg, MD, USA) and tested with a one-step RNA PCR kit, using primers specific for the *nsp2* gene (forward primer 5′-AGACCAGATGGAGGAGGATCTGC-3′; reverse primer 5′-AGTCGATGATGGCTTGAGCTGAG-3′). In the 60-day infection group, 42.9 (12/28) and 46.7% (14/30) of the fetuses examined were infected at 7 and 14 dpi, respectively. In the day 90 infected group, 41.7 (10/24) and 60% (12/20) of fetuses were positive for HP-PRRSV at 7 and 14 dpi, respectively. In the litters that were aborted at 13 and 17 dpi, the fetal infection rates were 63.6% (7 of 11 fetuses) and 50% (5 of 10 fetuses), respectively. Sows infected with HP-PRRSV at 60 days of gestation had a similar proportion of infected fetuses: 16 of 25 (64%) fetuses were infected at 21 dpi compared with 14 of 24 (58%) fetuses from sows infected at 90 days of gestation. These results demonstrate that fetuses from the 60-day and 90-day infected groups did not differ in their fetal infection rates.

Another way to evaluate the capacity of HP-PPRSV for placental infection is to determine the viral load in the fetal organs. The organs (spleen, thymus, lung, liver, heart, and kidney) of all fetuses were tested for the virus with qRT–PCR and their viral loads were compared among groups (Figure 2). The spleens of fetuses in the day 60 infected group contained significantly more virus than those from fetuses in the day 90 infected group at 7 and 21 dpi. At 14 dpi, the thymuses of fetuses in the day 60 infected group contained significantly more virus than those from fetuses in the day 90 infected group. Significantly more virus was also found in the hearts of the fetuses from the day 90 infected group at 7 dpi relative to the day 60 infected group, and in the kidneys of the fetuses from the day 60 infected group at 14 dpi relative to the day 90 infected group. The viral loads were significantly higher in the livers of the day 90 infected group fetuses than in the day 60 infected group at 7 dpi, but much lower at 21 dpi. The viral load in the fetal lungs did not differ in between groups at any time point. The statistical significance of inter-group differences were determined by two-way ANOVA analysis performed using GraphPad Prism 5.

**Figure 4:**
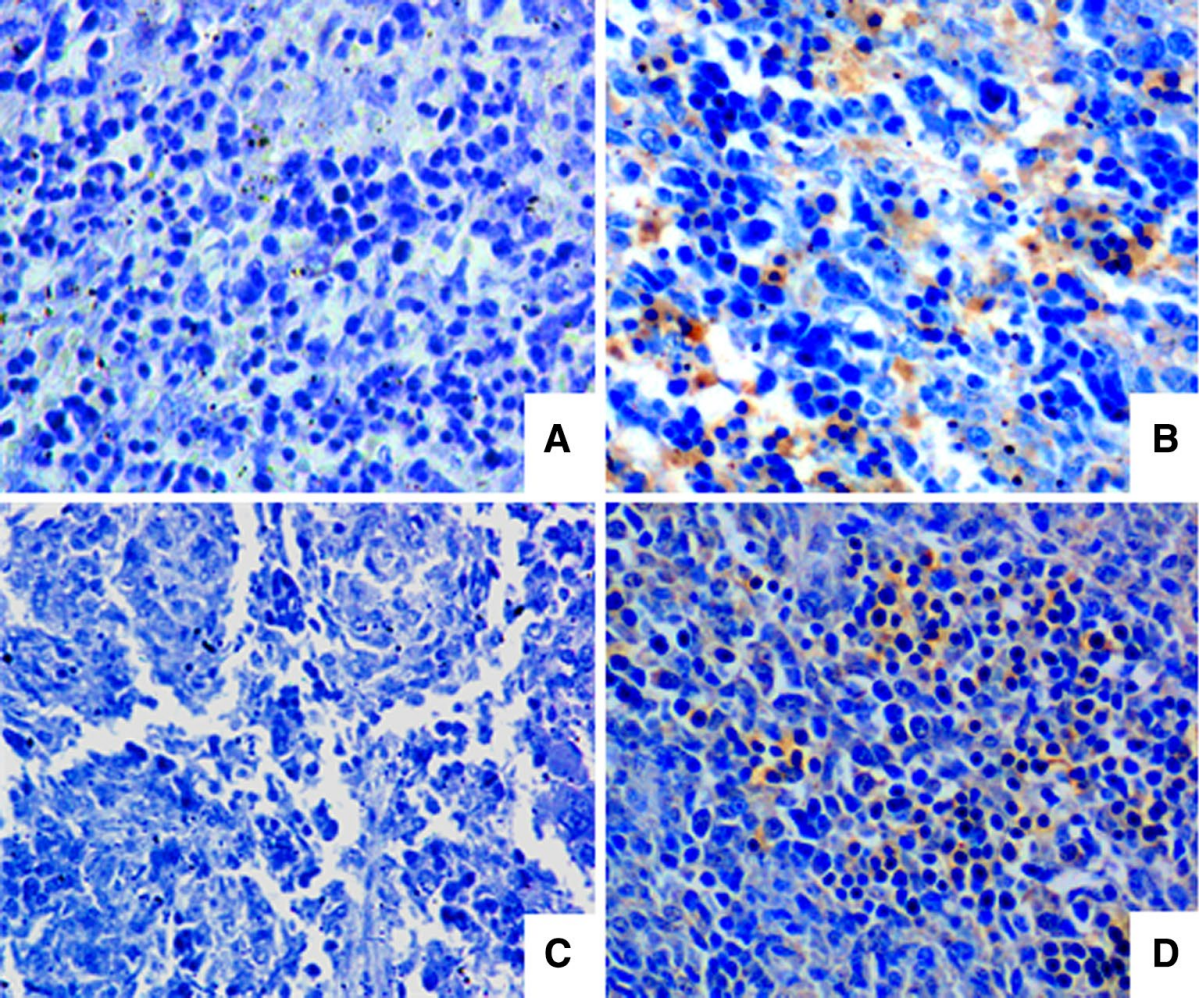
**Immunohistochemical detection of HP-PRRSV in the fetal spleen at 7 dpi.** (**A**, **C**) Negative control splenic tissue, in which mouse IgG was substituted for the primary antibody; (**B**) spleenic tissue from the day 60 infected group; (**D**) spleenic tissue from the day 90 infected group. All images are shown at ×40 magnification.

To confirm fetal infection, the spleens from the fetuses with high titers of HP-PRRSV were sectioned and viral antigen was detected with immunohistochemistry using the monoclonal antibody 6D10 (IgG1) directed against the PRRSV N protein [22]. Uninfected material stained with the monoclonal antibody 6D10 and infected material stained an anti-green fluorescent protein, a monoclonal antibody with the same isotype was used as the negative control. PRRSV-positive cells in the spleens of the infected fetuses appeared brown due to the presence of diaminobenzidine (Figure 4), but no PRRSV-positive cells were seen in the control fetuses.

## Discussion

The ability of PRRSV to cross the placenta and infect fetuses varies with strain. Successful transplacental infection depends on the ability of the virus to cross the placenta and replicate within the fetus, evading both maternal and fetal immune responses. Our results show that HP-PRRSV transplacentally infected fetuses in both the day 60 and 90 infected groups. Evaluation of fetal infection rates and viral loads in fetal organs revealed that HP-PRRSV is equally capable of crossing the placenta at day 60 and 90 of gestation.

Fetal infection rates at each time point were not significantly different and the viral loads were similar between the day 60 and 90 infected groups. These results support the hypothesis that fetuses are similarly susceptible to infection with HP-PRRSV at day 60 and 90 of gestation. Although in this study we did not simultaneously evaluate transplacental infection with classical PRRSV, previous studies have shown that the number of fetuses infected with classical PRRSV during mid-gestation is lower than the number infected during late gestation [14, 17, 18]; this is different from the result we obtained with HP-PRRSV.

In this study, much higher levels of virus were detected at 7 dpi in the placentas of sows infected at day 90 of gestation than in those from sows infected at day 60 of gestation; this is likely due to the lower expression of the Sn on placental macrophages at day 60 of gestation [21]. However, HP-PRRSV infection up-regulated the expression of Sn mRNA; Sn plays a vital role in receptor-mediated endocytosis of PRRSV. Low Sn expression may explain why the HP-PRRSV load was high in the day 90 placenta while the proportion of fetuses infected was similar to that seen in sows exposed on day 60. Of course, unknown interactions between HP-PRRSV and the host immune response may also explain these findings.

In the field, HP-PRRSV infection is complicated by concomitant infection by other pathogens, which can result in severe morbidity and mortality. In the present study, a lower abortion rate (10%) was observed in the day 90 infected sows. The reason for this lower rate may be their reduced opportunity for exposure to other pathogens under laboratory conditions, which would have improved the pregnant sows’ ability to fight the HP-PRRSV infection. In this single-pathogen model, we have provided experimental evidence that HP-PRRSV is equally capable of crossing the placenta and causing fetal infection in sows at day 60 and 90 of gestation. The severity of the disturbance to reproductive hormone concentrations caused by HP-PRRSV during late gestation may explain why only sows in late gestation aborted during this study [22].
